# Ex situ heart perfusion: A novel platform to test cardiovascular therapeutics in human hearts

**DOI:** 10.1016/j.jhlto.2025.100336

**Published:** 2025-07-03

**Authors:** John Onsy Louca, Magnus Althage, Marco Öchsner, Aravinda Page, Joao Pedro Nunes, Catherine Wilson, Sanjay Sinha, Simon Messer, James Matt Bae, Mostin Hu, Nicole Asemota, Sarah Fielding, Sai Bhagra, Neil Henderson, Johnny Lindquist, Daniela Später, Anna Collén, Kaushik Sengupta, Benjamin Challis, Justin Perkins, Stephen Large

**Affiliations:** a Jeffrey Cheah Biomedical Centre, Cambridge Biomedical Campus, The University of Cambridge, Cambridge, Cambridgeshire, UK; b Wellcome–MRC Cambridge Stem Cell Institute, Cambridge Biomedical Campus, The University of Cambridge, Cambridge, Cambridgeshire, UK; c Department of Medicine, The University of Cambridge, Cambridge, Cambridgeshire, UK; d Translational Science & Experimental Medicine Department, Research and Early Development, Cardiovascular, Renal and Metabolism (CVRM), Biopharmaceuticals R&D, AstraZeneca, Gothenburg, Sweden; e Royal Papworth Hospital, Cambridge, Cambridgeshire, UK; f Department of Pharmacology; The University of Cambridge, Cambridgeshire, UK; g Golden Jubilee Hospital, Glasgow, UK; h Integrated Bioanalysis Department, Clinical Pharmacology & Safety Sciences, AstraZeneca, Gothenburg, Sweden; i Bioscience Cardiovascular Department, Research and Early Development, Cardiovascular, Renal and Metabolism (CVRM), Biopharmaceuticals R&D, AstraZeneca, Gothenburg, Sweden; j Research and Early Development, Cardiovascular, Renal and Metabolism (CVRM), Biopharmaceuticals R&D, AstraZeneca, Gothenburg, Sweden; k Alliance Management, Business Development and Licensing, Biopharmaceuticals R&D, AstraZeneca, Gothenburg, Sweden; l Translational Science & Experimental Medicine Department, Research and Early Development, Cardiovascular, Renal and Metabolism (CVRM), Biopharmaceuticals R&D, AstraZeneca, Cambridge, UK; m Royal Veterinary College, Hertfordshire UK

**Keywords:** Ex-Situ Heart Perfusion, Cardiomyopathy, Drug discovery, Human model of heart failure, Angiogenesis, mRNA therapies, Vascular endothelial growth factor-A

## Abstract

**Background:**

Explanted hearts from recipients undergoing cardiac transplantation may be utilized as a human model of cardiomyopathy. Ex-situ perfusion of hearts allows control of the physiological and biochemical conditions of perfusion. *AZD8601 is* a novel modRNA for VEGF-A that was shown to be safe in a Phase IIa clinical trial – the EPICCURE trial. This proof-of-concept study aimed to demonstrate the potential utility of testing novel therapies on explanted recipient hearts using ex situ machine perfusion.

**Methods:**

In order to ascertain the expression of VEGF-A in a human model of cardiomyopathy, *AZD8601* was injected at high- and low-dose into the mid-myocardium of the left ventricle of human hearts explanted at the time of cardiac transplantation and perfused on the m0rgan, a novel, normothermic organ perfusion machine. Hearts were perfused ex situ for 6 h. After which, injection sites were biopsied and divided into subendocardium, mid-myocardium and sub-epicardial myocardium. Immuno-analysis was performed to assess levels of VEGF-A protein.

**Results:**

There were elevated levels of VEGF-A protein in the subendocardium and mid-myocardium of injection sites which received *AZD8601*. Low-dose and high-dose *AZD8601* resulted in a significantly higher concentration of VEGF-A protein in the myocardium.

**Conclusions:**

This study builds on the EPICCURE study, a phase IIa clinical trial which demonstrated safety of this mRNA in patients undergoing coronary artery bypass grafting. This study provides a novel model of diseased human heart for experimental studies utilizing ex situ heart perfusion.

## Graphical abstract

**Figure:**
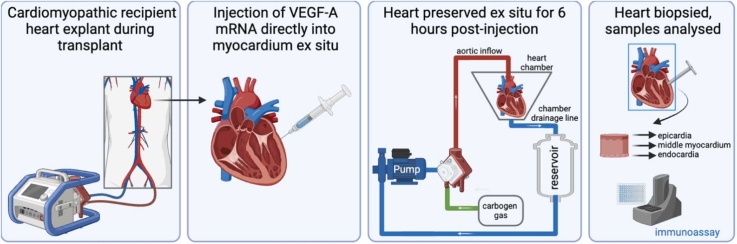
Graphical abstract. This summarises the methodology of the experiment.

## Introduction

Ex Situ Heart Perfusion (ESHP) was first developed in 1895 by the German physician Oscar Langendorff, and it has been one of the most extensively used isolated organ research models. Over the years it has resulted in countless crucial discoveries that have formed the basis of our understanding of heart physiology, biochemistry and pharmacology.^1^ In recent years there has been a revival of the technique for use in heart transplantation. The technique has enabled the development of non-heart-beating donation programs and has greatly increased transplant activity.^2^, ^3^

**Figure 1:**
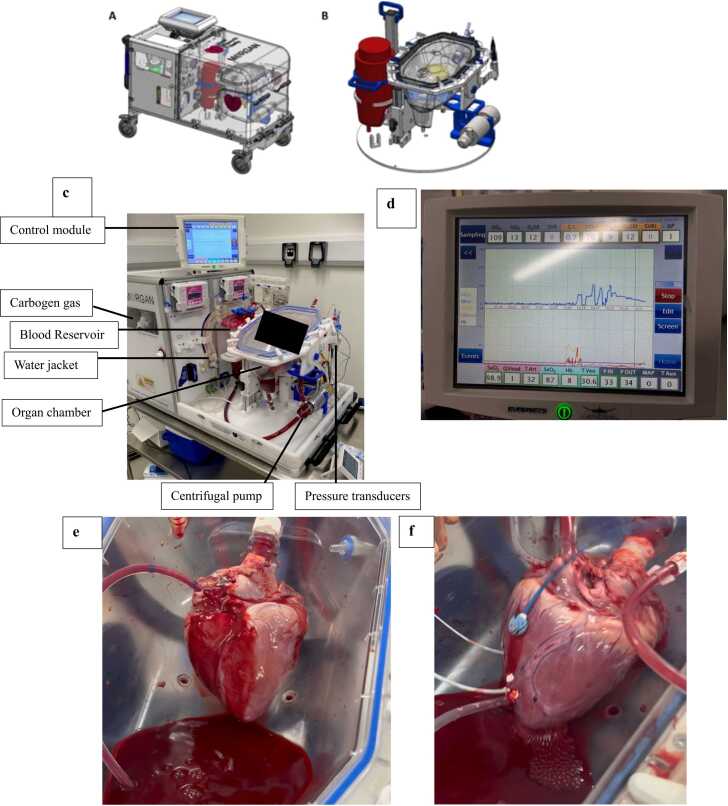
– The morgan components and set-up with a pig heart model. (a) The m0rgan device – a portable ex-situ heart perfusion machine. (b) the m0rgan disposable – with the cradle set only. The heart is placed in the organ chamber and mounted onto the machine via the aorta. (c) Annotated picture of the m0rgan device with a heart perfused ex situ. The organ chamber is detachable, and with additional tubing can be placed inside a MRI scanner, whilst the metallic frame is left outside. This requires additional perfusate to prime the circuit but offers a MRI compatible method to assess heart function. This photo does not show the oxygenator or flow probes. (d) The control screen on the m0rgan. It provides information on the delivery of oxygen to the tissue, pressures and flows at various points in the circuit. (e) the explanted heart of a pig on the m0rgan, in non-working mode – that is with an empty left ventricle, being perfused anterogradely via the aortic root. There is an LV vent in-situ, that prevents over distension of the left ventricle. (1f) an explanted pig heart in working mode – with blood entering via the left atrium, passing though the mitral valve and being pumped out by the left ventricle via the aorta. A pressure-volume loop catheter can be seen apically and a doppler US probe, used to measure tissue flow can be seen at the base of the left ventricle. These enable characterisation of blood flow at the tissue level as well as systolic and diastolic function ex-situ.

Human ESHP is particularly expensive. Currently, the only clinically available normothermic perfusion system is the Organ-Care-System (OCS), developed by Transmedics.^4^ Therefore, widespread use of the OCS beyond heart transplantation is not financially viable. There have been isolated reports of using the OCS to test therapies in a porcine model, however this model has not been widely replicated.^5^ We have developed an organ perfusion machine – the multi-organ perfuser (m0rgan) (Figure 1a-d) which is a continuous-flow pump device that can support the heart in either working or non-working mode (Figure 1e-f – demonstrating both working and non-working mode in a pig heart model). The cost of the m0rgan is significantly lower and enables human ESHP use to be expanded for research purposes.

Here, we use explanted human hearts from patients undergoing cardiac transplantation to test expression of vascular endothelial growth factor (VEGF-A) protein using a novel mRNA, *AZD8601* in diseased target tissue. *AZD8601* was shown to be safe in 7 patients undergoing coronary artery bypass grafting (CABG) in the EPICCURE study - a phase IIa clinical trial. In addition to this *AZD8601* has shown efficacy in large animal work and human non-myocardial tissue, however it has yet to be shown to be efficacious in human myocardium.^6^, ^7^, ^8^ Additionally there is no data on the distribution of protein within the human myocardium or optimal dose to maximize protein production.

There is therefore a need to test the efficacy of this mRNA in human myocardium. This study was a proof-of-concept study that aimed to demonstrate the utility of explanted recipient hearts, preserved ex situ as a model to test novel therapeutics.

## Methods

Ethical approval for the study was granted by the UK Wales research ethics committee (REC), Reference Number: 20/WA/0257.

Patients were excluded if they had undergone previous cardiac surgery. The explant of hearts from patients who have undergone previous cardiac surgery is complicated by the presence of adhesions, thereby potentially placing additional mental strain on the implanting surgeon. Only those patients on the cardiac transplant waitlist who provided informed consented to donate their explanted hearts to this study were included.

Three diseased hearts from patients undergoing cardiac transplantation (66-year-old male with ischemic cardiomyopathy, 40-year-old female with dilated cardiomyopathy and a 21-year-old female with hypertrophic cardiomyopathy) were explanted according to standard clinical practice. These hearts were immediately cooled and arrested with St Thomas’ 2 solution – a hyperkalaemic cardioplegic solution at 4 C to reduce the metabolic demand.

**Figure 2:**
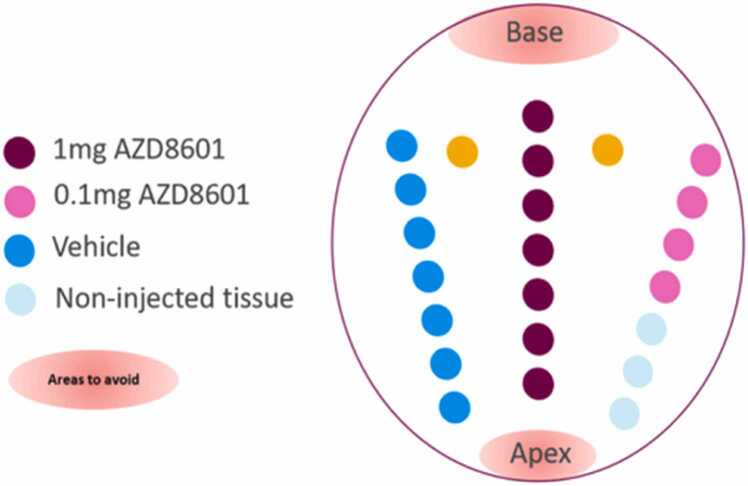
Example injection map of LV free wall. Left ventricle (LV) free wall injection map in Heart 1**.** High dose (1 mg), low dose (0.1 mg) mRNA and sodium citrate (Vehicle control) was injected into the free wall separated by 1 cm. LV free wall was chosen as it was readily accessible. Where there was additional space on the LV free wall, a sterile needle was placed into the LV, but no solution injected through it. The back up sites (yellow, not listed in figure) were used to sample non-injected LV. These were only taken if there was sufficient space in the LV free wall. These sites were both used as additional negative controls. Hearts 2 and 3 were injected in a comparable manner.

In order to attach the explanted heart to the m0rgan a Hemoshield® vascular graft was attached end-to-end to the aorta. The hearts were kept in cold cardioplegic solution for a mean of 73 min whilst the injections into the hearts were performed, and the aortic graft attached. The left ventricular free walls of these hearts were injected with either high-dose *AZD8601* (1 mg), low-dose *AZD8601* (0.1 mg) or a control solution of sodium citrate into the mid-myocardium (Figure 2). Direct injection was performed due to the short half-life of *AZD8601.* Moreover, this was the method of delivery performed in the EPICCURE trial. Injection sites were 1 cm away from each other, in order to maximize the number of injection sites, whilst also minimising contamination between sites. Injections were performed to a depth of 5 mm and injected over 30 s. The needle was held in place for a further 30 s after the injection to reduce the amount of injectate pushed out during systole, through the fine channel created by the needle.

Hearts were mounted onto the m0rgan and perfused anterogradely with a blood based perfusate for 6 h. The perfusate was composed of the following: 2 units of red blood cells, 500 ml Albumin 5%, 1 g Meropenem, 1 g Vancomycin, 200 mg Voriconazole, 50 ml Mannitol 10%, 5000 units of Epo Alpha, 20 mmol Sodium Bicarbonate 8.4% and 250 mg Methylprednisolone. Perfusates were composed whilst the hearts were prepared for mounting on the m0rgan.

The aortic root pressure maintained for the duration of perfusion varied between 50 and 70 mmHg with flows between 500 and 750 ml/min, at a temperature of 34 °C. None of the hearts demonstrated rising aortic root pressures in spite of a constant coronary flow rate. This indicated that these hearts, free of any indication of ‘no-reflow’ during reperfusion, had an intact endothelium throughout the experiment. A left ventricle vent was inserted through the left atrium and mitral valve, to prevent left ventricle distension injury secondary to trace aortic regurgitation and thebesian flow.

Oxygen saturation was 96–99% and the pCO2 was between 5 and 7 kPa This was achieved with a flow of carbogen (CO2 enriched compressed air) of 200 ml/min. Hearts were paced at 100 beats per minute throughout the duration of the experiment. The left ventricle was kept beating, in non-working mode (i.e. an unloaded, empty ventricle). Blood gases were performed every 30 min to monitor electrolytes, pH and lactate levels. Glucose levels were maintained at >5 mmol, K+ was maintained between 3 and 5mmol and Ca2+ was maintained at 0.8–1.2mmol. After this, hearts were removed from the m0rgan and trans-mural punch-biopsy samples of injection sites were collected and further divided into sub-endocardium, mid-myocardium and sub-epicardial myocardium. Samples were frozen in liquid nitrogen and transferred to Gothenburg for VEGF-A protein analysis.

Quantification of human VEGFA protein in tissue samples was conducted using an immunoassay for human VEGFA (V-PLEX Human VEGFA Kit, K151RHD-1, Mesoscale Diagnostics) in combination with human VEGFA 165 protein (Recombinant Human VEGFA 165 protein, 293-VE-010, R&D Systems) used for the calibration standards and quality controls (QCs).

Statistical differences between the injected and non-injected groups for each layer of the heart, were assessed by the Kruskal-Wallis test, a rank-based, non-parametric test, due to the non-normal distribution of the data. Analysis between groups was carried out using Dunn’s multiple comparisons tests, a similarly rank-based test, to identify between which groups significant differences exist. Analyses were carried out using the scipy library in Python 3.9. Figures were generated using Prism 10.

## Results

### Histopathology analysis

Heart 1 was explanted from a 66-year-old man who suffered from ischemic cardiomyopathy. Histology demonstrated extensive replacement fibrosis involving the anterior wall of both ventricles, septum and lateral left ventricular wall – in keeping with a left main stem infarct.

Heart 2 was explanted from a 40-year-old woman with dilated cardiomyopathy. Histology showed left widespread, predominantly mid-myocardial zone replacement fibrosis with some associated interstitial fibrosis in the left ventricle.

Heart 3 was explanted from a 21-year-old woman with hypertrophic cardiomyopathy. On histological analysis, there was clear myocyte disarray in the left ventricle – particularly in the LV septum. However, there was no significant fibrosis.

### Protein expression in myocardium

**Figure 3:**
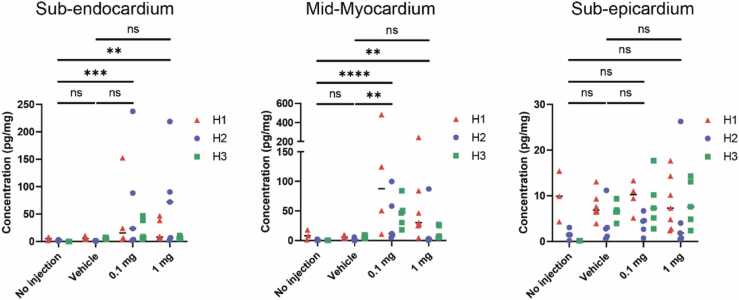
– VEGF-A protein concentration in the three layers of heart tissue. VEGF-A protein expression in the sub-endocardium, mid-myocardium and sub-epicardial myocardium across 3 hearts (heart 1 (H1) red triangles, heart 2 (H2) blue circles and heart 3 (H3) green squares). P<0.01**, P<0.001***, P<0.0001.

Fig. 3 shows the concentrations of VEGF-A protein across the 3 layers of myocardium. Table 1 demonstrates the mean VEGF-A protein concentration across the 3 hearts. Table 2a, Table 2b demonstrate the results of the Kruskal Wallis statistic and Dunn’s multiple comparison tests. No difference in VEGF protein expression was observed in sub-epicardial samples. Some endocardial samples had clear increased VEGF A protein expression, but concentration of VEGF A varied considerably across samples, 21% (3/14) of the 0.1 mg treated group and 18% (3/17) of the 1 mg treated group contained greater than 50 pg/mg VEGF A concentration. Endocardial samples in both the high-dose and low-dose *AZD8601* exhibited significantly increased expression of VEGF-A protein compared to control injections (p=0.0297 in the low-dose group, p=0.021 in the high-dose group).

**Table 1:** Mean & Median VEGF-A concentration levels in myocardium stratified by area

Injection	Tissue	Mean Level of Protein Expression (SD), (pg/mg Tissue)	Median Level of Protein Expression +/- [IQR], (pg/mg Tissue)
0.1 mg	endo	18.7 (24.8)	6.8 +/- [20.6]
0.1 mg	mid	34.8 (32.1)	24.2 +/- [40.3]
0.1 mg	epi	6.9 (4.7)	5.2 +/- [6.1]
1 mg	endo	32.2 (54.7)	8.2 +/- [32]
1 mg	mid	25.3 (28)	25.0 +/- [24.2]
1 mg	epi	8 (7.1)	4.9 +/- [10.7]
Citrate buffer	endo	4.8 (2.8)	5.2 +/- [5.3]
Citrate buffer	mid	5.6 (2.7)	4.8 +/- [4.7]
Citrate buffer	epi	6.2 (3.4)	6.6 +/- [3.7]

A summary of mean protein concentration in (pg/mg tissue) of all 3 hearts used in this experiment. Means are +/- standard deviation

**Table 2a:** Kruskal Wallis for VEGF-A concentration levels between endocardial, mid-myocardial samples and epicardial samples across the 3 hearts

VEGF-A Protein	endo	mid	epi
Kruskal-Wallis statistic	7.8488	16.85	0.1975
p-value	**0.0089**	**0.0002**	0.9060

Kruskal-Wallis statistic demonstrating a significant difference in the distribution of VEGF-A expression in endocardial and mid-myocardial sections.

**Table 2b:** Dunn’s multiple comparison test between control, low dose and high dose VEGF-A injections

Heart Layer				
Endocardial		CS vs 0.1 mg	CS vs 1 mg	0.1 mg vs 1 mg

	Dunn’s multiple comparison test	−13.03	−12.94	0.08403
	p-value	**0.0297**	**0.021**	>0.99

Mid-myocardial		CS vs 0.1 mg	CS vs 1 mg	0.1 mg vs 1 mg

	Dunn’s multiple comparison test	−20.73	−9.088	11.64
	p-value	**0.0001**	0.1749	0.0635

Epicardial		CS vs 0.1 mg	CS vs 1 mg	0.1 mg vs 1 mg

	Dunn’s multiple comparison test	−2.244	−1.059	1.185
	p-value	>0.99	>0.99	>0.99

Testing between groups is performed using Dunn’s multiple comparison test. There is a significant difference in VEGF-A concentration in endocardial samples between the citrate buffer and both the low dose and high dose mRNA. There is also a significant difference in VEGF-A concentration in mid-myocardial samples between the citrate buffer and low dose mRNA.

The mid-myocardium samples had the greatest VEGF A protein expression and tissues injected with 0.1 mg of mRNA showed a significantly increased protein expression when compared to vehicle control (p<0.001). Variability was again seen across samples 7/14 (50%) showing greater than 50 pg/mg VEGF concentration. No significant difference was observed following 1 mg injection (p=0.17), 3/17 (18%) of samples recoding greater than 50 pg/mg VEGF concentration. The protein expression data for each individual biopsy sub-section is available in Supplemental Tables 1a-c.

### Discussion

This proof-of-concept study demonstrates the potential utility of ex situ machine perfusion in testing the efficacy, dosing and distribution of novel therapies. The EPICCURE trial, a phase IIa trial, demonstrated that *AZD8601* was safe and resulted in potential improvements in quality of life in 7 patients undergoing coronary artery bypass grafting.^6^ Previous work with longer experimental trajectories has demonstrated that *AZD8601* increases blood flow in a pig heart model of myocardial infarction and in forearms of type 2 diabetic patients.^7^, ^8^ This study demonstrates expression of this mRNA in cardiomyopathic hearts. It complements the findings of the EPICCURE study which trended towards an improvement in quality of life in patients that received *AZD8601*, however further cases are needed to better understand the optimal dose or distribution of *AZD8601.* Further studies using the model may be able to effectively demonstrate differences in VEGF-A expression in different forms of cardiomyopathy. This work has the potential, to inform trial recruitment and inform the target population for this intervention, given that expression appeared to be higher in dilated and ischemic cardiomyopathic hearts compared to hypertrophic cardiomyopathic hearts. However, further cases would be needed before any definitive conclusions can be drawn.

### Expression of AZD8601

This proof-of-concept study demonstrates for the first time that diseased human mid-myocardium tissue can be promoted to drive specific protein expression from a therapeutic mRNA. The sub-epicardial myocardium displayed no VEGF-A protein expression across all samples while the sub-endocardium displayed a variable response, which may be due to the lack of mRNA retention at, or diffusion/delivery to peripheral heart sites. Alternatively, it may be that the vascular flow and the downward movement of the injectate preferentially delivered the mRNA to the sub-endocardium instead of the sub-epicardium, given that coronary blood flow travels from epicardium to endocardium. The higher expression of the VEGF-A in the subendocardium may be of increased clinical benefit given the susceptibility of the subendocardium to ischemia.^9^ We believe that there was little diffusion of *AZD8601* between injection sites, evidenced by the very low VEGF-A protein levels in control sites, adjacent to *AZD8601* sites.

Comparison of mRNA doses revealed that low-dose AZD8601 led to a significant increase in VEGF-A protein in the mid-myocardium compared to vehicle-injected or non-injected sites within the hearts (Fig. 3**b**). High-dose AZD8601 displayed more variable results, showing no significant difference in protein expression to vehicle treatment. The reasons for this difference are not immediately clear. It may be that higher mRNA concentrations lead to stress-induced inhibition of translation, while lower doses of mRNA allow for optimal protein expression. Nevertheless, the differing dose responses highlight the unique use of ESHP to determine the efficacy, distribution and dosing of novel cardiovascular therapeutics directly in target diseased human tissue.

There was also considerably variability in the production of VEGF-A, even within the same heart for the same dose. The reasons for this are not clear. It may represent poor uptake of the mRNA by the myocardium. Alternatively, it may be possible that injection samples near arterioles may have resulted in the injectate being washed away before *AZD8601* could translocate into the myocardium. Further work is needed to better understand the variability in VEGF-A levels.

From this limited study, it would appear that *AZD8601* is expressed in higher levels in dilated and ischemic cardiomyopathic hearts compared to hypertrophic cardiomyopathy. This may be linked to the degree of fibrosis in these hearts. Both the ischemic and dilated cardiomyopathic hearts demonstrated higher levels of fibrosis than the hypertrophic heart on histological analysis and both expressed higher levels of VEGF-A protein. However, given the fact that there was only a single heart with each pathology this cannot be confidently claimed. Further use of this model, and clinical studies are necessary to better understand the effects of *AZD8601* in different cardiomyopathies.

### Advantages of this model

Whole organ perfusion offers several advantages over other human models such as cell culture, organoids and sliced-organ preparations.^10^ Whole organ perfusion enables organ function to be investigated more comprehensively – with the opportunity for imaging, functional and biochemical parameters to be utilized to measure heart function. The m0rgan is able to be placed into a MRI scanner, and with small modifications to the circuit hearts, can be perfused in working mode, which in turn would enable pressure-volume (PV) loop data to be generated.^11^ PV loop is the gold standard for assessing heart function ex-situ, given its ability to assess heart function in a load independent manner. We have previously demonstrated the utility of PV loops in the assessment of heart function in DCD donor hearts.^12^

Besides this, established biomarkers of heart function, including lactate and troponin can be used accurately in ESHP unlike in established myocardial slice preparations, where the mass of myocardium is not sufficient for biomarker use*.*^13^ This study used lactate as a proxy of adequate heart perfusion. Future studies will use imaging and functional tools to better assess heart function during perfusion and provide novel insights into the effects of therapeutics on heart function.

Ex-situ organ perfusion could additionally facilitate the exploration of multiple different treatments, to better understand therapeutic combinations with synergistic effects. In the case for *AZD8601*, possible synergistic co-treatments include mRNA-based therapies to promote cardiomyocyte replication or stem cell therapies.^14^, ^15^ This would theoretically enable appropriate revascularisation of regenerated myocardium, which may be a particularly attractive method to repair damaged hearts. This is particularly exciting, given recent advancements in stem cell therapies, with long-term engraftment of stem cell patches, demonstrating maturation and engraftment up to 3 months later in a patient with end-stage heart failure.^16^ Recent work has suggested that the beneficial effects of stem cells may be mediated by chemokine signaling and an inflammatory, wound-healing response.^17^

Turned down donor-livers, preserved on ex situ perfusion machines have demonstrated the potential efficacy of novel cholangiocyte organoids.^18^ This study, as well as our study presented here, demonstrate the great potential of ex-situ perfused human organ models, unsuitable for transplantation or explanted from recipients.

Ninety percent of medicines used in clinical trials fail, 50% of these fail due to a lack of efficacy.^19^ This pre-clinical model of human end-stage heart failure could provide valuable insight into human efficacy of medicines at early stages of the drug discovery pipeline, well before expensive clinical trials commence. Furthermore, using transplant patients discarded hearts poses minimal ethical issues and no risk to patients. Each year there are 9000 heart transplants globally. These hearts represent a significant resource of end-stage diseased hearts as a model for testing novel biological therapies.

### Limitations of this study

This study is limited in part by the small sample size. Only three explanted recipient hearts were used to test the efficacy of *AZD8601*. Whilst this small sample size demonstrated a clear increase in VEGF-A protein levels, a larger sample size would better enable the increase in protein level to be assessed. This study also used hearts with 3 different kinds of cardiomyopathy (dilated, ischemic and hypertrophic). This limits the utility of comparisons of results between different hearts in this study. Moreover, this study is also limited by the lack of functional data. Whilst biochemical data was regularly measured using a portable blood gas analyser, this study did not measure functional data on heart systolic or diastolic function. As mentioned above, this should be implemented in future studies to better understand the effects of novel therapies on heart function in the acute and sub-acute phase.

### Limitations of the model currently

Normothermic perfusion time is limited, and thus currently limits the utility of our model to understanding rapid drug kinetics, as is the case for mRNA. Future work using ESHP should aim to prolong perfusion times. Normothermic perfusion times have been reported up to 12 h, and our own lab has perfused healthy porcine hearts for 15 h.^20^ We believe that it will be possible to prolonged ex-situ perfusion time enabling studies on therapeutic efficacy and allow functional assessment to be performed. It would be exciting to assess cell integration of emerging cell-based approaches for cardiac regeneration directly in target tissue in the future.

## Conclusion

ESHP represents a novel and underexplored model for end stage heart failure enabling the testing of new therapeutics in a human setting. This proof-of-concept study effectively showcases the efficacy of this model. Close collaboration between advanced heart failure centres, transplant authorities, academics in the regenerative space and the pharmaceutical industry will be essential to maximizing the utility of these explanted organs.

## Source of Funding

This study was funded in its entirety by AstraZeneca.

## Disclosures

JOL, MÖ, AP, JPN, SM, JMB, MH, CHW, NA, SF, SL have nothing to declare.

MA, NH, JL, DS, AC, KS, BC are employees of AstraZeneca.

SS is a founder and equity holder in ABS Biotechnologies GmbH.

## Declaration of Competing Interest

The authors declare that they have no known competing financial interests or personal relationships that could have appeared to influence the work reported in this paper.

## Footnotes

Appendix A Supplemental data associated with this article can be found in the online version at doi:10.1016/j.jhlto.2025.100336.
