# Supplementary material for: Ex situ heart perfusion: A novel platform to test cardiovascular therapeutics in human hearts
Source: JHLT Open. 2025 Jul 3;9:100336. doi: 10.1016/j.jhlto.2025.100336 (PMC12310391; doi:10.1016/j.jhlto.2025.100336)
Supplement: Supplementary file 1 — Supplemental material [file mmc1.docx]

**Supplementary Ex situ heart perfusion: a novel platform to test cardiovascular therapeutics in human hearts**

**Supplementary Table 1a – Protein Concentrations in subendocardium**

|  | **No Injection** | **Citrate Saline** | **0.1mg** | **1mg** |
| --- | --- | --- | --- | --- |
| Heart 1 | 5.26 | 5.37 | 3.51 | 8.16 |
|  | 8.26 | 5.23 | 24.32 | 4.87 |
|  | 1.61 | 7.09 | 152.77 | 6.52 |
|  |  | 3.65 | 6.86 | 6.83 |
|  |  | 7.63 |  | 38.50 |
|  |  | 5.48 |  | 8.69 |
|  |  | 11.02 |  | 46.89 |
|  | | | | |
| Heart 2 | 1.10 | 1.10 | 1.69 | 90.25 |
|  | 3.31 | 1.60 | 23.55 | 6.98 |
|  | 1.17 | 1.84 | 3.61 | 2.25 |
|  | 1.96 | 1.63 | 88.36 | 218.92 |
|  |  | 0.87 | 237.45 | 72.22 |
|  | | | | |
| Heart 3 | 0.20 | 5.37 | 6.83 | 5.48 |
|  | 0.20 | 5.23 | 38.50 | 11.02 |
|  | 0.20 | 7.09 | 8.69 | 8.16 |
|  | 0.20 | 3.65 | 46.89 | 4.87 |
|  |  | 7.63 | 3.51 | 6.52 |

Supplementary Table 1a. Protein concentrations in subendocardium (pg/mg tissue)

**Supplementary Table 1b – Protein concentrations in mid-myocardium**

|  | **No Injection** | **Citrate Saline** | **0.1mg** | **1mg** |
| --- | --- | --- | --- | --- |
| **Heart 1** | 8.0 | 9.19 | 50.59 | 4.45 |
|  | 17.86 | 4.13 | 483.17 | 25.02 |
|  | 1.02 | 3.17 | 124.54 | 27.10 |
|  |  | 4.76 | 10.84 | 46.44 |
|  |  | 9.34 |  | 84.13 |
|  |  | 5.47 |  | 30.20 |
|  |  | 7.47 |  | 243.10 |
|  | | | | |
| **Heart 2** | 2.14 | 0.2 | 8.67 | 87.2 |
|  | 0.20 | 0.2 | 99.75 | 1.33 |
|  | 0.20 | 1.88 | 4.68 | 1.55 |
|  | 0.20 | 1.73 | 11.72 | 3.18 |
|  |  | 5.79 | 58.3 | 0.20 |
|  | | | | |
| **Heart 3** | 0.20 | 9.19 | 46.44 | 5.47 |
|  | 0.20 | 4.13 | 84.13 | 7.47 |
|  | 0.20 | 3.17 | 30.20 | 4.45 |
|  | 0.20 | 4.76 | 18.12 | 25.02 |
|  |  | 9.34 | 50.59 | 27.10 |

Supplementary Table 1b. Protein Concentrations in the mid-myocardium (pg/mg tissue)

**Supplementary Table 1c – Protein concentrations in epicardium**

|  | **No Injection** | **Citrate Saline** | **0.1mg** | **1mg** |
| --- | --- | --- | --- | --- |
| **Heart 1** | 9.91 | 6.43 | 5.17 | 2.39 |
|  | 15.45 | 9.37 | 9.47 | 4.87 |
|  | 4.33 | 6.89 | 11.04 | 14.34 |
|  |  | 6.56 | 13.33 | 10.19 |
|  |  | 3.96 |  | 7.30 |
|  |  | 13.05 |  | 17.70 |
|  |  | 7.62 |  | 2.78 |
|  | | | | |
| **Heart 2** | 3.06 | 0.65 | 0.74 | 0.51 |
|  | 1.46 | 2.80 | 4.47 | 1.83 |
|  | 1.48 | 1.13 | 4.59 | 0.77 |
|  | 0.20 | 3.06 | 6.71 | 4.04 |
|  |  | 11.20 | 2.68 | 26.3 |
|  | | | | |
| **Heart 3** | 0.2 | 6.43 | 10.19 | 13.05 |
|  | 0.2 | 9.37 | 7.30 | 7.62 |
|  | 0.2 | 6.89 | 17.70 | 2.39 |
|  | 0.2 | 6.56 | 2.78 | 4.87 |
|  |  | 3.96 | 5.17 | 14.34 |

Supplementary Table 1c – Protein concentration in epicardium (pg/mg tissue).
